# Foreign Bodies in Lower Urinary Tract: Case Report and Review of Literature

**DOI:** 10.5812/ircmj.7074

**Published:** 2013-07-05

**Authors:** Praveen Kumar Pandey, Amit Goel, Dilip Kumar Pal, Anup Kumar Kundu

**Affiliations:** 1 Department of Urology, SSKM Hospital, Kolkata, India

**Keywords:** Foreign Bodies, Lower Urinary Tract Symptoms, Urinary Tract Infections

## Abstract

Foreign bodies in lower urinary tract may present in a different number of ways. We report four cases of such unusual presentation. Physical examination and plain radiograph was sufficient enough to confirm our diagnosis in all cases. The cases belonged to different age groups and three out of four cases were managed by open surgical approach. One foreign body was removed using cystoscope. Prompt surgical management prevented urinary tract infections and long term complications in these patients.

## 1. Introduction

The foreign bodies in lower urinary tract could be of various origins ranging from iatrogenic to self-introduced. The correct possible management could avoid significant morbidity in these patients. The objective of this case report is to highlight unusual presentation of foreign bodies in lower urinary tract with different aetiologies and their possible management.

## 2. Case Presentation

### 2.1. Case No 1

**Figure 1.**
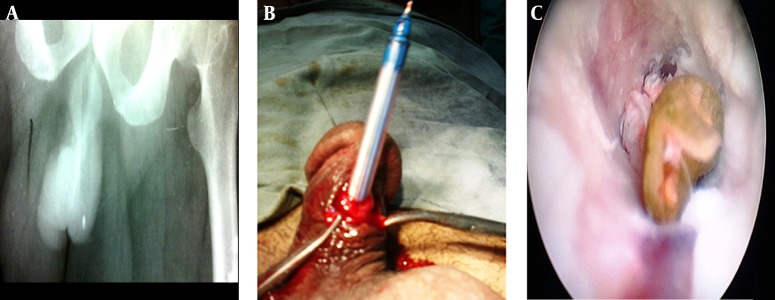
A) Plain radiograph showing a radiolucent shadow of foreign body over penile shaft. B) A ball pen seen protruding out of urethrotomy incision. C) Endoscopic image showing wooden stick in unhealthy urethra.

A 60 year man presented to the emergency department with alleged complaint of somebody forcibly introducing something into his urethra after making him unconscious. On examination, few drops of blood were present over the meatal opening and bladder was palpable just above the symphysis pubis. A long cylindrical object was felt occupying almost entire penile urethra. The X-ray of pelvis confirmed our findings (Figure 1A). A ventral urethrotomy was done over the impending area of rupture of the penile skin and a long ball pen was removed (Figure 1B). Patient was catheterised and wound closed in layers. The catheter was removed on tenth post-operative day. The patient is having no complaints till date and is on regular follow up. 

### 2.2. Case No 2

A 40 year male was known case of urethral stricture disease and had undergone visual internal urethrotomy (VIU). He was on irregular urethral calibration. So, in due course of time the stricture got apparently tough. Finding it difficult to negotiate the stricture with the soft catheter, the patient chose to try a harder pipe, a wooden stick. He gave it a try and missed the stick in urethra. Urinary retention was relieved by performing a suprapubic cystostomy (SPC). Urethroscopy showed a foreign body in penile urethra (Figure 1C) and was removed using alligator forceps. The patient was catheterised per urethra. Finally, a routine surgery was planned for the definitive management of the urethral stricture disease. 

### 2.3. Case No 3

**Figure 2.**
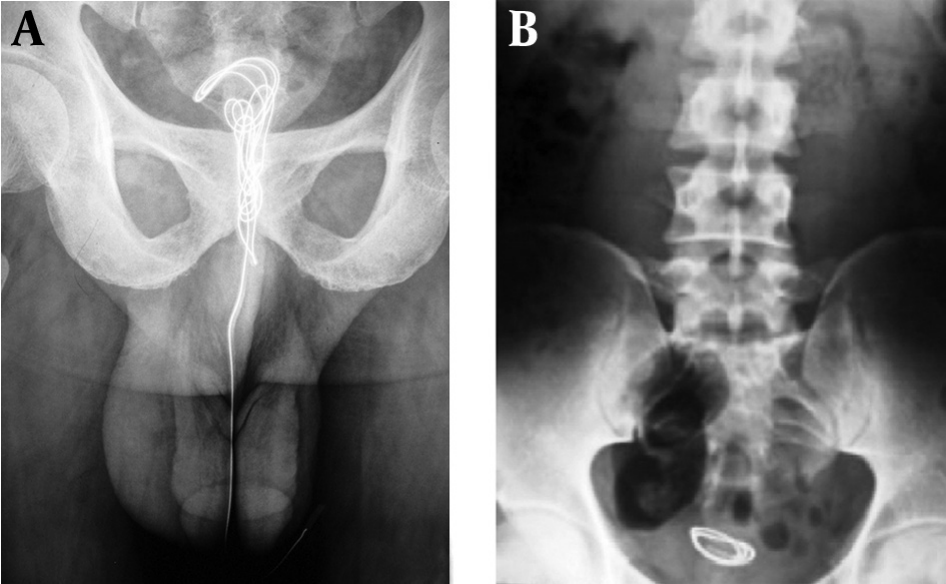
A) Plain radiograph showing a coiled wire in bladder and extending in urethra outside the meatus; B) Plain radiograph showing a coiled wire in urinary bladder.

A 25 year young male presented to the emergency department with a wire protruding from his meatal opening. He revealed that in order to achieve sexual gratification he tried to introduce a metallic wire in his urethra. As he found no resistance, he kept on introducing it further. Suddenly he realised that he had pushed a long portion of the wire inside his urethra. Any attempt to take it out failed, as the wire had coiled inside his bladder. The wire was palpable in urethra and X-ray KUB confirmed our findings (Figure 2 A). 

An emergency cystostomy was done and wire delivered out of the bladder. A per-urethral catheter was placed after closing the bladder.

### 2.4. Case No 4

A 38 year male presented to us with recurrent episodes of hematuria and bilateral inguinal discomfort. Physical examination was unremarkable. The X– ray KUB showed a foreign body in his bladder (Figure 2 B). Looking at the film, he promptly recognized it to be a metallic wire which he had placed in his bladder one month back to achieve sexual gratification. Wire was removed by emergency cystotomy and per urethral catheter was placed. 

## 3. Conclusions

Foreign bodies in lower urinary tract have always been a topic of joke in urology and other disciplines of surgery. They could present as an emergency or could be discovered accidentally while finding a cure for long standing urological symptoms. Some patients may even choose to ignore their symptoms as it may not hamper their lifestyle significantly. Self-introduced foreign objects are found in those suffering from mental disorders (1). However, it is also reported in patients of different age groups as a result of some sexual misadventure (2). Such patients may be hesitant to seek medical help until their symptoms force them to do so. These objects may also be iatrogenic in origin. The incidence of iatrogenic foreign bodies in lower urinary tract seems to be increasing with a rise in surgical procedures being performed in these anatomical areas. Rarely, these may perforate into urinary bladder after migrating from the adjoining organs or anatomical sites (3). Even leeches may enter the urinary bladder per urethra and may present as an unusual cause of haematuria (4). These are found in both genders with an increased frequency in females. The possible explanation lies in the fact that females have a shorter and a relatively straight urethra. On the contrary, here we find all the cases to be males as females in developing countries like ours seem to be still neglecting their health.

These foreign bodies may cause recurrent urinary tract infections, haematuria, urinary bladder perforations, encrustations, stone formation or even pelvic abscess. They may be diagnosed by detailed history taking, a focused physical examination, X-ray KUB or if required, ultrasonography of pelvis. In three of the discussed cases, X-ray KUB confirmed the diagnosis. However, one case required endoscopic examination for it. Some of these foreign bodies can be managed endoscopically whereas others may require open surgical approach. This approach may allow their en mass removal or at times fragmentation of these objects prior to their removal in small pieces. Use of nephroscope sheaths, pnuemovesicoscopy and even holmium laser has been reported as an effective armamentarium in endoscopic management of these foreign bodies (5, 6). We were able to remove only one of these foreign bodies by endoscopic approach whereas rest of the cases required open surgery. In conclusion, these lower urinary tract foreign bodies may have various presentations. The concerned urologist or surgeon must have a significant suspicion to diagnose them at an early stage. At first, a minimal invasive approach might be chosen. But, one must not hesitate to adopt open surgical approach if endoscopic measures fail or these facilities are not available at the concerned centre.

*Our Research work was not at all sponsored by any particular firm or a profit making organization*.
